# Explaining factors affecting help-seeking behaviors in women with urinary incontinence: a qualitative study

**DOI:** 10.1186/s12913-020-06047-y

**Published:** 2021-01-13

**Authors:** Fahimeh Rashidi Fakari, Sepideh Hajian, Soodabeh Darvish, Hamid Alavi Majd

**Affiliations:** 1 School of Nursing and Midwifery, Shahid Beheshti University of Medical Sciences, Tehran, Iran; 2 Midwifery & Reproductive Health research centre, School of Nursing and Midwifery, Shahid Beheshti University of Medical Sciences, Tehran, Iran; 3 Department of Obstetrics & Gynaecology, Fellowship of Female Pelvic Floor Medicine and Reconstructive Surgery, Shahid Beheshti University of Medical Sciences, Tehran, Iran; 4 Department of Biostatistics,School of Paramedicine, Shahid Beheshti University of Medical Sciences, Tehran, Iran

**Keywords:** Urinary incontinence, Help -seeking behaviors, Qualitative research

## Abstract

**Background:**

Urinary incontinence is widely accepted to be among the most important issues in the global health system. However, only a limited number of women are referred for treatment because different factors complicate help-seeking behaviors. The aim of this study was to explain the factors affecting help-seeking behaviors in women suffering from urinary incontinence.

**Methods:**

This study was a qualitative study using the conventional content analysis approach. The study was conducted from December 2018 and August 2019 in Tehran, Iran. The participants were 34 women with urinary incontinence selected using a purposive sampling method. The content analysis approach was based on the Graneheim and Lundman method, and qualitative data management software was used for analysis.

**Results:**

Data analysis illustrates two themes; “ facilitator “ and “ inhibitor “; the categories “not perceiving disease”, “shame”, “ negative support of important others”, and “non-optimal health care system” were among the inhibitors and the categories “ reduced quality of life “ and “ positive support of important others” were found to be facilitators of help-seeking behaviors.

**Conclusions:**

The findings of the present study highlight the need for understanding the underlying facilitators and inhibitors of help-seeking behaviors in women with urinary incontinence. We suggest that healthcare providers consider an open dialogue with patients and consider their subjective beliefs and life context during routine visits to facilitate early diagnosis of the disease and ultimately lead to an improvement in the woman’s quality of life.

## Background

According to the International Continence Society, any complaint of involuntary leakage of urine is called urinary incontinence (UI ( [1]. UI is currently one of the most important and significant global health system issues [2].

Women are susceptible to involuntary leakage of urine due to their urinary system anatomy [3]. Moreover, some studies reported the relationship between demographic and midwifery factors such as age, parity, and delivery method with urinary incontinence [4–6].

The prevalence of UI varies because of the diversity in definitions and diagnostic methods [7]. A review study conducted in different parts of the world (North America, Asia, Europe, Africa, Australasia), reported an average UI prevalence of 27.6% in women (range: 4.8–58.4%) [2]. A study in the Canada, also demonstrated that one in five women experience UI during their lifetime [8]. Overall prevalence of UI in the Iranian women is 46% [9].

The physical effects of incontinence on the body include rash, persistent skin irritation, and bacterial and fungal infections. Anxiety, stress, and depression are said to be the psychological consequences of incontinence. Moreover, UI disrupts normal daily activities and the social presence of the patient. In addition, involuntary leakage of urine also disrupts sexual intercourse [10–12]. Consequently, UI has an undeniable detrimental effect on patient quality of life, which emphasizes the important role of treatment in the improvement of the patient’s quality of life [10, 13, 14].

There are various and effective treatment options for urinary incontinence. Behavioral treatments, pelvic floor muscle exercises, various medications, and surgery are the most common treatment strategies [15]. However, a limited number of women seek help, according to one study, merely 22% of women sought treatment for urinary incontinence [16].. Moreover, most women with UI try to manage it themselves rather than seeing a physician for treatment, which in turn, worsens related complications [17].

There are a number of studies, as in the north of England and Sweden, dealing with the reasons not help-seeking for treatment or delayed seeking [18, 19]. In the north of England, a study found that one reason for women’s reluctance to help-seeking was the attribution of incontinence to natural changes such as those caused by aging [19]. In Germany and Denmark, another study reported that help-seeking behaviors depended on the intensity of the complications caused by incontinence [20].

Women’s experiences with UI in Iran indicate a tendency among Iranian women to tolerate the inconvenient conditions caused by incontinence without seeking help [21]. However, with regard to the relatively high prevalence of urinary incontinence in Iranian women [9], no study has been done to discover the factors affecting UI treatment. Therefore, considering the high prevalence of UI in Iranian women, it is necessary to increase knowledge about effective and comprehensive factors help-seeking behaviors. By recognizing those factors, it may be possible to facilitate help-seeking and diagnosis to ultimately reduce the prevalence of UI in women in the community.

The concept of help-seeking behaviors in the field of health refers to planned behavior aimed at getting help from professional healthcare providers on the detection of changes in health status. Help-seeking behaviors are complex behaviors influenced by a variety of factors and are consequently subject to change in different contexts [22]. Therefore, we can reduce the time between the onset of the problem and receiving professional help by determining the effective factors at play. In other words, early diagnosis and timely treatment could be achieved by identifying the factors affecting the patients’ help-seeking behaviors. In formation on such behaviors can then be transferred to healthcare providers. Hence, we aimed to assess the factors affecting help-seeking behavior in women with UI in Iran.

## Methods

### Study design

This qualitative research was the first study conducted exclusively on the Iranian population to determine the factors affecting the pursuit of treatment. We chose a qualitative approach because we wanted to understand the underlying nature of the issue, and for this reason, we must investigate the views of individuals based on their personal experiences.

### Settings, sample and recruitment

The research context was the urology clinics (urology clinics for convenient access to women seeking help for UI) of teaching hospitals of Shahid Beheshti University of Medical Sciences, and also, public places (parks, mosques, etc.) (for access convenient to women not seeking help) or in other parts of the city on interviewee request. Purposeful sampling was performed. The participants of the study included women with urinary incontinence who met the inclusion criteria as follows: they were willing to be interviewed, were not pregnant, were suffering from urinary incontinence (any kind of incontinence (for at least 6 months or more, had been treated or had avoided referral for treatment, did not suffer from of any acute mental disorders or disease that could interfere with the interview, and were able to communicate verbally with the researcher in Persian. On the other hand, if the interviewee was not willing to continue participation at any time during the interview or if she was unwilling to disclose her experiences, she was excluded from the study.

After reviewing the inclusion criteria, the participants of the study included 34 women with urinary incontinence with an average age of 54.50 years (range: 29–75 years). The minimum and maximum duration of urinary incontinence in the participants were 1 year and 25 years; also, the lowest and highest number of childbirths were zero (0) and 11 times, respectively.

### Data collection

Data collection methods included in-depth interviews with women with UI from December 2018 to August 2019. The samples were taken in the research context, after the evaluation the inclusion criteria, and approval of the diagnosis of UI by Bradley’s Questionnaire for Urinary Incontinence Diagnosis (QUID). The questionnaire has previously been published elsewhere [23] (see also Online Supplementary file 1: Study questionnaire (Bradley’s questionnaire)). The interviews were conducted individually and recorded using a digital voice recorder with the permission of the participants. All interviews were conducted in person by the same interviewer (researcher). The interviews began with an open-ended question (What are the reasons for your referral or non-referral for the treatment of UI?) and continued with the probing questions (Please explain more. What do you mean? etc.) or clarification statements (What you said means ......., You meant that ....) were used during the interviews. Because of the maximum diversity (achieved by selecting people from different places, levels of education, employment, and income by a demographic questionnaire), data saturation did not occur until interview 34, in other words, no new information was obtained in interview 34. The duration of the interviews was determined by the participants and their willingness to continue the interview, but the sessions took on average 30–60 min. At the end of each interview, the researcher listened to the recorded interviews several times and then verbatim transcribed them into a Microsoft Word file.

### Data analysis

We conducted conventional content analysis approach based on principles recommended by Graneheim and Lundman (2004), which included the implementation of each interview after it ended, reading the entire text to fully understand its contents, determining meaning units and initial codes, classifying similar initial codes into more comprehensive classes, and determining the main theme (latent content) (Table 1: an example of analysis process). In this method, classes and their names were created from data [24]. MAXQDA software (Ver.10) was used for analysis.

**Table 1:** An example of analysis process

Meaning unit	Code	Sub-Subcategories	Subcategories	Main Category	Theme
I change my clothes regularly, if I am not home I change both my underwear and my pants when I come (P. 5).	Frequent change of clothes	Adaptation to symptoms	Self-control	Not perceiving disease	Inhibitor
I go to the bathroom frequently so that my bladder is empty so that I do not urinate (P. 8).	Frequent urination				
When I want to go out, I always take a sanitary pad (P. 9).	Use of sanitary pad				
I try not to drink water or tea (P.1).	Limited fluid intake	Changing eating habits			
I follow a diet of fruits, vegetables, and herbs (P. 2).	Eating healthy food				
I was told that you have a prolapse bladder, but I do not, because if I had, I would have felt the prolapse of the bladder (P.8).	Lack of information the nature of the disease	Unawareness of the nature of the disease	Unawareness		
Where I worked, I used the well water; it was near a gas station, and people said the gasoline was leaking into the well. When we used water, it had a bad smell, and after that, I developed this urinary problem (P.1).	Wrong information about the cause of the disease				
Women with the disease don’t pursue treatment because it has no treatment. I don’t know if there is a cure (P. 2).	Unawareness of about the curability of the disease	Unawareness of treatment			
I understand that bladder lift surgery is useless because it is not something that holds the bladder (P.16).	Misconceptions about how to treat				

### Rigor and trustworthiness

Lincoln and Guba’s (1985) trustworthiness criteria, which include credibility, dependability, conformability, and transferability, were used to increase the rigor of the collected data [25]. Credibility included prolonged engagement, reviewing by participants and feedback, and requesting a review by the members of the research team. Two external researchers were asked to review the data to ensure its dependability. Moreover, code-recode method (for 2 interviews) was used for at least 2 weeks, in this way two interviews were coded, then 2 weeks later the same interviews were re-coded. The coding was very similar both times. In addition, the confirmability of the findings was verified by two auditors familiar with qualitative research. The transferability of the findings was confirmed by selecting participants with the highest ability to communicate and by maintaining diversity in the selection of participants. Moreover, the authors strived to provide thick descriptions for those who seek to transfer the results.

### Ethical considerations

The research protocol and sampling has been approved by the ethics committee at Shahid Beheshti University of Medical Sciences (the code: IR.SBMU.PHNM. 1397.33) in the Tehran (Iran). Written informed consent was obtained from each woman included in the study.

## Results

After reviewing the participants’ perspectives on the factors affecting their help-seeking behaviors, two themes were obtained. In the process of analyzing and comparing data after categorizing codes and eliminating similar codes, 60 codes, 36 sub-sub-categories,17 sub-categories, 6 main categories, and 2 themes (facilitator and inhibitor) were extracted (Table 2). The inhibitor theme included the main categories of “not perceiving disease “, “shame”, “ negative support of important others “, and “ non-optimal health care system” and the facilitator theme included “ reduced quality of life “ and “positive support of important others”.

**Table 2:** Classification of Theme, main categories and subcategories

Sub-Subcategories	Subcategories	Main categories	Theme
Attributing to natural processes	Non-acceptance incontinence as a disease	Not perceiving disease	Inhibitor
Non-warning nature of incontinence			
Adaptation to symptoms	Self-control		
Changing eating habits			
Unawareness of the nature of the disease	Unawareness		
Unawareness of treatment			
Fear and worry investigation of the disease	Fear- worry		
Fear of invasive treatments			
Concealment of the disease	Shame related to the nature of incontinence	Shame	
Shame of expressing to caregivers			
Shame of talking about genital area	Shame related to the genital area		
Shame of observation of genital area			
Cost of diagnostic	Enormous costs	Non-optimal health care system	
Cost of therapeutic			
Inaccessibility	Poor quality of care		
Unavailability			
Defective reference system			
Inappropriate behavior of caregivers			
Providing incorrect information	Negative effect on decision-making	Negative support of important others	
Dissuade from visiting			
Reverse therapeutic experiences	Creating doubts about treatment outcomes		
Treatment as ineffective			
Misconceptions in the family	Role of family deterrence		
Numerous expectations from a woman			
Lack of perception of the spouse’s problem	Role of husband deterrence		
Lack of spouse support			
Encouragement to refer	Positive effect on decision-making	Positive support of important others	Facilitator
Recommend treatment places			
Expressing experiences of improving	Transfer positive therapeutic experiences		
Confirm of non-invasive treatments			
Emotional support	Support		
Financial support			
Intensity increase of symptoms	Exacerbation of the disease	Reduced quality of life	
Symptoms of accompanying weakening			
Limitations	Pervasiveness of the disease		
Exhausted			

### Inhibitor theme

#### Not perceiving disease

Not perceiving disease was one of the reasons for not seeking help that it consists of unawareness, not accepting incontinence as a disease, fear- worry and self-care.

Not accepting incontinence as a disease was related to its attribution to natural processes, as well as the absence of warning signs. In this regard, one participant said, “*Incontinence is normal for those who are getting older*” (Participant 5, age 64, mixed type).

“*Anyway, we have given birth many times; eventually, incontinence is related to many pregnancies and deliveries …* “ (Participant 13, age 75, mixed type).

*“I have urinary incontinence, but I don’t have any pain or bleeding at all … “* (Participant 31, age 44, mixed type).

Some participants controlled the disease by adapting to its symptoms and changing their eating habits. One participant said, “*I follow a diet of fruits, vegetables, and herbs”* (Participant 2, age 75, urgency type).

“*I try not to drink water or tea*” (Participant 1, age 31, mixed type).

Unawareness of the nature of the disease (as the cause of disease genesis) and unawareness of its treatment prevents people from making the right decision in dealing with it. As one participant stated:“ *Where I worked, I used well water; it was near a gas station, and people said the gasoline was leaking into the well. When we used water, it had a bad smell, and after that, I developed this urinary problem*” (Participant 1, age 31, mixed type).

“*Women with the disease don’t pursue treatment because it has no treatment. I don’t know if there is a cure*” (Participant 2, age 75, urgency type).

Fear and worry about the consequences of the disease, as well as fear of invasive treatments, also affected referral rates.

“*I think women are afraid to go to a physician for their disease because of being diagnosed with a dangerous disease*” (Participant 28, age 54, urgency type).

“*If a doctor tells me to have surgery, I won’t do it; why should I put myself at the mercy of the surgeon’s knife?*” (Participant 13, age 75, mixed type).

#### Shame

In some participants, the shame of having UI led to hiding the disease and not telling the problem to healthcare professionals.

“*I didn’t tell anyone about my problem; it’s not a matter to be talked about ...”* (Participant 3, age 67, mixed type).

“*I’m embarrassed .... it’s so hard .... to go to the doctor and say I’m incontinent; that I can’t hold it … “* (Participant 17, age 50, stress type).

Another part of the shame was the embarrassment of exposing the genital area. Participants were ashamed of being examined by their caregivers and even talking about it. This embarrassment became more apparent in relation to male healthcare specialists, so they preferred same-sex caregiver.

***“****I told myself that if I went to the doctor, he might want to examine me; he would look down there (the genital area), which I wouldn’t allow* “ (Participant 13, age 75, mixed type).

“*I didn’t go to see the doctor; I took medicine myself; I’m embarrassed to talk about a problem in the genital area …*” (Participant 24, age 48, urgency type).

After realizing the presence of male students in the doctor’s room, one participant said, “*I came here to be examined by a female doctor, but the men examined me; her students were male; they’re the ones examining the patients”* (Participant 21, age 69, mixed type).

#### Non-optimal health care system

Diagnostic and therapeutic costs were important in the use of medical services because many people are unable to afford them.

“*The medicine is expensive; my husband told me to, tell the doctor to prescribe medicine that was covered by insurance. I said, ‘What can I do? “* (Participants 23, age 50, mixed type).

“*I heard that this hospital is free, so I came here ... I have health insurance. I just paid for the commute”* (Participant 9, age 61, urgency type).

“*I just came for a check-up, but they did a lot of tests, an ultrasound; they exhausted me …*. *I paid a lot of money*” (Participant 31, age 44, mixed type).

Inaccessibility to services in some areas, unavailability (for example, long waiting time), lack of patient referral because of the defective referral system, and inappropriate behavior (as negligence, disrespect) of caregivers were some of the poor quality of services that participants complained about. One participant said:

*.... They [care providers] said that I should do the urodynamic test whose device is not available here; they told me to go to … [Province center]*” (Participant 27, age 55, mixed type).

“*If I didn’t have the necessary time, I would go to private centers; now that I have the time, I came here (public hospital). You have to wait a long time for your turn*” (Participant 14, age 48, stress type).

“*One says do surgery, another one says no, the other one says go to that clinic, another one says go to this doctor; they give addresses, this is better, that’s better; I don’t know where to go, what to do*” (Participant 18, age 42, urgency type).

One of the patients, complaining of the disrespectful treatment said, “*Excuse me, but some people insult us; for example, my belly is big, one of them said ‘What a big belly,’ and ‘Why is your belly so big?’ They insulted me repeatedly, for this reason. I didn’t like that hospital ...; that’s why I didn’t go to that hospital anymore*” (Participant 23, age 50, mixed type).

“*I’m a patient of Dr...., but her students always examine me. She is there too, but she doesn’t answer me; she doesn’t pay attention to the patient at all*” (Participant 4, age 63, urgency type).

#### Negative support of important others

Important others had a dual effect (negative and positive) on the patients’ lives. In the inhibitor theme, negative support of important others by incorrect information about the disease and dissuade the patient from visiting had a negative effect on a person’s decision to seek help. Furthermore, the expression of reverse therapeutic experiences and ineffective treatment can cause doubt about the consequences of treatment and therefore, prevent help- seeking.

“*My sister-in-law has been suffering from urinary incontinence for almost 6 years. She says, it’s because of the cesarean, and that I will get better; she tells me not to go to the clinic, I’ll get better, it’s a complication of surgery”* (Participant 26, age 42, urgency type).

“*My friends say we have the same problem, one of my friends had surgery and says she still has the problem; she tells me not to do it and that it’s useless*” (Participant 10, age 69, mixed type).

“*One of my daughters said that her mother-in-law had this problem, so she had surgery; the doctor pierced her bladder during the operation, and now, instead of a few drops, she has become completely incontinent”...* (Participant 23, age 50, mixed type).

In the inhibitor theme, the role of the family and spouse in referring for treatment was considerable. On the one hand, misconceptions in the family affected help-seeking behaviors, and on the other hand, the various expectations and responsibilities of the woman in the family prevented her from paying attention to her own problem. Moreover, her husband’s lack of perception about the problem, as well as his lack of support, delayed seeking help. The women said:

“*My children, my daughter-in-law, and my son-in-law shouldn’t know about my incontinence problem. If they find out, they think I’m loose. It’s ugly for me. If I want to see a doctor, I have to lie*” (Participants 33, age 51, stress type).

“*My family thinks that I’m lax*” (Participant 8, age 52, urgency type).

“*Look, I did the urodynamic test two years ago. I haven’t been able to show it to a doctor yet. I have a handicapped child at home and a lot of work to do; I’m so busy*” (Participant 15, age 60, mixed type).

“*My husband says ‘Can’t you go to the bathroom sooner?’ I’m under a lot of pressure involuntarily, I can’t hold myself; I can’t control it*” (Participant 4, age 63, urgency type).

“*At least I don’t have financial problems, but what about other women?!! They’re financially dependent on their husbands, so they do not have the authority to see a doctor whenever they wanted* “ (Participant 33, age 51, stress type).

### Facilitator theme

#### Positive support of important others

In the facilitator theme, others encouraged patients to see a doctor, as well as suggesting places to get treatment, that had a positive effect on the decision to seek help. Another way others facilitated help-seeking behaviors was by providing positive treatment experiences. Therefore, speaking about signs of recovery after receiving treatment, as well as transferring the experience of non-invasive treatments pursued patients to use treatment. The supports were also a stimulus for help-seeking. Participants said:

“*My gynecologist told me to follow-up for the urinary problems, and my sister confirmed it, she said go visit a doctor, follow-up your problem* “ (Participant 19, age 35, stress type).

“*My mother also has this problem, she went to see a doctor; the doctor prescribed pills for her; she says she’s better … I said to myself, why did I bother myself when I could get better with one pill!! “* (Participant 20, age 62, urgency type).

“*My husband isn’t like some men who don’t pay attention to their wives. If I have surgery, I’m not worried because he does everything for me*” (Participant 32, age 47, stress type).

#### Reduced quality of life

Reduced quality of life was one of the factors that were extracted as a facilitator for help-seeking from the participants’ views. As the symptoms worsened, the limitations, and exhaustion from the disease, increased the chances of seeking help.

“*It wasn’t so bad before; it’s gotten worse for the past couple of months. When I get out of bed to go to the bathroom in our bedroom before I take three steps, I lose control of my urine”* (Participant 21, age 69, mixed type).

*“I go to the bathroom a lot, that’s why I get wet all the time; my body is constantly burning”.*

(Participants 15, age 60, mixed type).

*“I couldn’t go out much; I didn’t go to parties, If that was not possible, I stayed there for just two or three hours, I was tired”* (Participant 23, age 50, mixed type).

“*I was obsessed with the bathroom, I was tired, I was looking for it everywhere I went … “* (Participant 15, age 60, mixed type).

## Discussion

In the present study, the main factors affecting help-seeking behaviors in women with UI were studied using a qualitative approach for the first time in Iran. Our findings indicated that there were different facilitating and deterring factors influencing the help-seeking behaviors and successful treatment.

The emergence of non-alarming nature of UI as a reason for not seeking help and also attributing incontinence to natural processes in the findings indicates a lack of proper perception about the nature of UI. As a result of the unrealistic perception about the nature of this disease, they did not have a correct perception of treatment either, and they assumed UI to be incurable, and with this assumption, some participants chose self-care strategies. Some patients did not go to see a doctor because of the fear and worry caused by the probable outcomes of further investigation of the disease or the fear of invasive treatments, which resulted from the lack of awareness. These results are consistent with other results reported on help-seeking behaviors in patients with UI, in which adaptation mechanisms, belief in the naturalness of incontinence, and unawareness of the treatment options were mentioned as the reasons for avoiding treatment [19, 26]. In this study, most patients avoided referring for treatment assuming that surgery was the only treatment for UI, while many of them may not have had the criteria for surgery at all; the fact that they thought surgery was the only cure also resulted from their low awareness. Women who were more aware of the nature of incontinence (such as the cause and treatment) were also more likely to help-seeking [27]. On the other hand, it should be noted that care providers may actually have a lower tendency to recommend supportive therapies; examining this possibility would require investigation into the financial motives of physicians, especially in terms of stakeholders-induced demands.

Shame was another main category of inhibitor theme in the study. In general, shame is mostly considered a cultural issue. As it only 3% of cases barrier to referral to treatment accounted in the United States, and it did not play a significant role in the help-seeking in the Netherlands [28, 29]. However, a sense of shame in most women was reported in Turkey [30]. Some participants in the study hid their incontinence and did not seek treatment out of shame because of the embarrassing nature of incontinence. Some women were even ashamed of mentioning the issue of incontinence with caregivers. It has also been found that women were ashamed to talk about their UI to their doctors [31], which could be because incontinence is considered to be a private issue, to the extent that it may even be a taboo; The sense of shame about UI is even more than the shame of the depression disease [32]. Another part of the shame is related to being uncomfortable with talking about UI and the examination of the genital area, especially if done by a male doctor, which could be one of the reasons for the unwillingness to be treated by a male care provider. Women with UI prefer to receive help from a female physician [30]. In one study, while female patients preferred to have a female physician in the field of obstetrics and gynecology, this preference was not reported in the case of choosing a family physician [33], reflecting the fact that gender preferences may be further seen in diseases that necessitate the examination of private areas of the body such as the genital area as in the case of UI.

Moreover, the main categories of the non-optimal health care system included cost and quality of care. Most participants mentioned the very high costs of diagnostic and therapeutic procedures as one of the factors delaying their pursuit of treatment, as a result of the lack of basic health insurance or insufficient coverage of diagnostic and treatment costs by the insurance companies. Therefore, they preferred not to refer for treatment or to postpone it as much as possible. Because, affordability is effective in receiving treatment [34]. Some participants in our study stated that some services were provided only in certain areas and people had to travel to access these services; consequently, inaccessibility caused some people to avoid seeing a doctor or to delay it as much as possible. On the other hand, sometimes services were accessible, but they were provided with delay or had a long waiting list. In other words, the availability of services was often poor. Furthermore, some patients were confused by the referrals, and as a result, they deviated from the main treatment path due to the defective service providence of the referral system. An efficient referral system would definitely lead to enhanced service quality [35]. Moreover, inappropriate behavior of caregivers was another aspect of service quality. In fact, how services were provided affected the quality of services, which in turn influenced patient satisfaction [36]. In the interviews of the present study, participants mentioned the care providers’ negligence, disrespect, and neglect of their dignity. The overcrowding of patients in health centers, especially in teaching and public centers because of their lower costs, reduced the amount of time and attention the patient received for their problem. On the other hand, focus on students’ clinical education led to the ignorance of respect and dignity, in particular, in private issues such as UI.

The undeniable role of the important others, such as relatives, friends, and spouse, was another finding of the present study; these people played both a deterring and facilitating role in the interviews. In one study, the relatives’ influence was one of the factors related to referral for UI [19]. This influence is so great that some people preferred to seek help from non-official sources such as relatives, which could be because of their greater accessibility [37]. Another reason may be the private and sensitive nature of the issue. For example, reports indicated that patients benefited from the help of others for their mental health problems [37, 38]. In this study, the transfer of positive experiences of relatives and their support acted as a facilitator, and the family had a special place in the interviews. However, there were concerns about seeking help from relatives, because such help may be harmful, prohibitive, or useless and thus would play a negative role in help-seeking [37]. According to this study, information, experiences, misconceptions, expectations, and lack of support from relatives had a deterring effect on women’s thoughts and performance. One of the significant misconceptions of the relatives was giving the “laxity” label to a person with UI. It seems that the “laxity” label had a lot of negative connotations in this society; this stigmatizing labeling forced the patient to hide her incontinence.

Moreover, the husband could have both a deterring and a facilitating role. Therefore, the spouse could be a deterring factor by not understanding the woman’s problem and not supporting her due to lack of intimacy. On the other hand, the husband’s sincere support for his wife played a facilitating role in some patients’ help-seeking behaviors. However, the spouse’s deterring role was more prominent, which could be attributed to the financial dependence of women, however, it is more likely to be related to men’s authority in this society.

Another major facilitating category of this study was the reduced quality of life. The exacerbation of the disease and its pervasiveness fell in this category, i.e. as the symptoms of disease worsened and invaded more dimensions of the patient’s life, the likelihood of help-seeking behavior increased. In other words, the reduced quality of life is accompanied by an increase in help-seeking. It should be noted that the patients’ quality of life changed from the onset of the disease, but they seek treatment only when these changes were tangible and intolerable. Various studies have reported the effect of UI on quality of life [10, 11, 18]. There is a reverse relationship between the quality of life and help-seeking behavior. For instance, a study showed that people with poorer quality of life had referred for treatment more [31], In other words, poor quality of life positively influences help-seeking behaviors [18]. The intensity of symptoms was also directly related to these behaviors. Indeed, patients with more severe symptoms sought more help [29]. However, extracting the reduced quality of life category as a stimulus for referrals indicated the importance of screening and diagnosis in the early stages of the disease.

In general, the extracted factors in this study, including; shame, not perceiving disease, reduced quality of life, were almost similar to the results of other studies, but factors non-optimal health care system and supportive effects of important others were different from previous findings.

The manifestation of the non-optimal health care system category in this study could be related to the inefficient implementation of the family physician plan in cities in this community. The improper implementation of the family physician plan and, as a result, the weakness of the referral system increases the number of referrals to specialized medical centers, imposes high costs, increase waiting time, and ignorance of respect and dignity. Finally, all of the above reasons lead to the creation of the non-optimal health care system as a category.

The negative support and positive support of important others categories in this study may be related to the family structure in this community. In this society, there are still a lot of family relations, so the mutual influence on individuals, both in a negative and a positive way, is undeniable.

### Strengths

One of the strengths and innovations of the study was the co-extraction of the factors affecting help-seeking for the treatment in both forms of facilitating and deterring factors. In addition, sampling was not limited only to those help-seeking or those who had not help-seeking behaviors or only to a specific age group; therefore, the results would be useful to healthcare providers because it can be generalized to a larger population.

Moreover, this study investigated the views of women with UI regarding the factors affecting their help-seeking behaviors for the first time in this society.

### Limitations

As in other qualitative studies, generalizability, which is typically higher in quantitative research, was one of the limitations of this study. However, we strived for maximum diversity in recruiting the participants, and various strategies were used to increase the study acceptability and objectivity. Hence, our findings seem to have acceptable reliability.

## Conclusions

This study determined the factors affecting help-seeking behaviors for the treatment utilization in women with UI in the form of two inhibitor and facilitator themes. Diagnosis and screening would be accelerated by introducing the effective factors extracted in this study to healthcare providers so that they may be considered in dealing with and treatment of women who refer to clinics or healthcare centers. For example, since UI is a private issue and care providers are aware of the women’s shame to express it, they can be the pioneers to examine the symptoms and perform the initial screening by respecting and maintaining one’s dignity with simple diagnostic methods (such as questionnaires), which do not require them to examine the patients. Furthermore, education the factors affecting help-seeking to healthcare providers, in particular, at the first level of the referral system, at in-service training and regular retraining programs, with an emphasis on maintaining patients’ dignity, could have a fundamental and significant effect on the changes in the attitudes and awareness, modify misconceptions and the health literacy level of society. The care providers should consider the support of relatives, in particular, the family, in designing therapeutic interventions to reduce both the negative burden of the disease and enable women to adjust their roles and more easily follow-up on the treatment of UI with the comprehensive support.

The final result, it is better to pay attention to people’s subjective beliefs and life context in routine care in an open dialogue with patients for early diagnosis disease.

## Data Availability

The datasets used and/or analyzed during the current study are stored in a file that is kept in the Vice-Chancellor for Research and are available from the corresponding author on reasonable request, but participants’ personal information is confidential and is not shareable.

## Abbreviation

UI: Urinary incontinence

## References

[1] Abrams P, Cardozo L, Fall M, Griffiths D, Rosier P, Ulmsten U (2002). The standardisation of terminology of lower urinary tract function: report from the Standardisation Sub-committee of the International Continence Society. Neurourol Urodynamics.

[2] Minassian VA, Drutz HP, Al-Badr A (2003). Urinary incontinence as a worldwide problem. Int J Gynecol Obstet.

[3] Weledji EP, Eyongeta D, Ngounou E (2019). The anatomy of urination: What every physician should know. Clin Anatomy.

[4] Kılıç M (2016). Incidence and risk factors of urinary incontinence in women visiting Family Health Centers. Springerplus.

[5] Agarwal BK, Agarwal N (2017). Urinary incontinence: prevalence, risk factors, impact on quality of life and treatment seeking behaviour among middle aged women. Int Surg J.

[6] Åhlund S, Rothstein E, Radestad I, Zwedberg S, Lindgren H (2020). Urinary incontinence after uncomplicated spontaneous vaginal birth in primiparous women during the first year after birth. Int Urogynecol J..

[7] Hunskaar S, Burgio K, Clark A, Lapitan M, Nelson R, Sillen U (2005). Epidemiology of urinary (UI) and faecal (FI) incontinence and pelvic organ prolapse (POP). Incontinence..

[8] Swanson JG, Kaczorowski J, Skelly J, Finkelstein M (2005). Urinary incontinence: common problem among women over 45. Can Fam Phys.

[9] Rashidi F, Hajian S, Darvish S, Alavi Majd H (2019). Prevalence of urinary incontinence in Iranian women: systematic review and meta-analysis. Iran J Obstet Gynecol Infertil.

[10] Trantafylidis SC-A (2009). Impact of urinary incontinence on quality of life. Pelviperineology.

[11] Asoglu MR, Selcuk S, Cam C, Cogendez E, Karateke A (2014). Effects of urinary incontinence subtypes on women's quality of life (including sexual life) and psychosocial state. Eur J Obstet Gynecol Reproductive Biol.

[12] Gomes TA, de Arruda Faber M, Botta B, Brito LGO, Juliato CRT (2020). Severity of urinary incontinence is associated with prevalence of sexual dysfunction. Int Urogynecol J.

[13] Kwon BE, Kim GY, Son YJ, Roh YS, You MA (2010). Quality of life of women with urinary incontinence: a systematic literature review. Int Neurourol J.

[14] Senra C, Pereira MG (2015). Quality of life in women with urinary incontinence. Revista da Associação Médica Brasileira.

[15] Vij M, Thomson A (2012). Management options for female urinary incontinence. Prescriber.

[16] Xu D, Wang X, Li J, Wang K (2015). The mediating effect of ‘bothersome’urinary incontinence on help-seeking intentions among community-dwelling women. J Adv Nurs.

[17] Park S, Yeoum S, Kim Y, Kwon HJ (2017). Self-management experiences of older Korean women with urinary incontinence: a descriptive qualitative study using focus groups. J Wound Ostomy Continence Nurs.

[18] Hägglund D, Walker-Engström ML, Larsson G, Leppert J (2001). Quality of life and seeking help in women with urinary incontinence: a population-based study. Acta Obstetricia et Gynecologica Scandinavica.

[19] Vethanayagam N, Orrell A, Dahlberg L, McKee KJ, Orme S, Parker SG (2017). Understanding help-seeking in older people with urinary incontinence: an interview study. Health Soc Care Commun.

[20] Pedersen LS, Lose G, Høybye MT, Jürgensen M, Waldmann A, Rudnicki M (2018). Predictors and reasons for help-seeking behavior among women with urinary incontinence. Int Urogynecol J.

[21] Pakgohar M, Hamid TA, Ibrahim R, DM V (2015). Elderly Community Dwelling Women's Experiences of Managing Strategies for Urinary Incontinence (UI): A Qualitative Research.

[22] Cornally N, McCarthy G (2011). Help-seeking behaviour: A concept analysis. Int J Nurs Pract.

[23] Bradley CS, Rovner ES, Morgan MA, Berlin M, Novi JM, Shea JA (2005). A new questionnaire for urinary incontinence diagnosis in women: development and testing. Am J Obstet Gynecol.

[24] Graneheim UH, Lundman B (2004). Qualitative content analysis in nursing research: concepts, procedures and measures to achieve trustworthiness. Nurse Educ Today.

[25] Lincoln YS, Guba E, Inquiry N (1985). Newbury Park. CA: Sage Publications.

[26] Lamerton TJ, Mielke GI, Brown WJ (2020). Urinary incontinence in young women: Risk factors, management strategies, help-seeking behavior, and perceptions about bladder control. Neurourol Urodynamics.

[27] Kim JS, Lee EH, Park HC (2004). Urinary incontinence: prevalence and knowledge among community-dwelling Korean women aged 55 and over. J Kor Acad Nurs.

[28] Dugan E, Roberts CP, Cohen SJ, Preisser JS, Davis CC, Bland DR (2001). Why older Community-Dwelling adults do not discuss urinary incontinence with their primary care physicians. J Am Geriatr Soc.

[29] Teunissen D, van Weel C, Lagro-Janssen T (2005). Urinary incontinence in older people living in the community: examining help-seeking behaviour. Br J Gen Pract.

[30] van den Muijsenbergh ME, Lagro-Janssen TA (2006). Urinary incontinence in Moroccan and Turkish women: a qualitative study on impact and preferences for treatment. Br J Gen Pract.

[31] Kinchen KS, Burgio K, Diokno AC, Fultz NH, Bump R, Obenchain R (2003). Factors associated with women's decisions to seek treatment for urinary incontinence. J Women Health.

[32] Elenskaia K, Haidvogel K, Heidinger C, Doerfler D, Umek W, Hanzal E (2011). The greatest taboo: urinary incontinence as a source of shame and embarrassment. Wiener Klinische Wochenschrift.

[33] Amir H, Tibi Y, Groutz A, Amit A, Azem F (2012). Unpredicted gender preference of obstetricians and gynecologists by Muslim Israeli-Arab women. Patient Educ Counsel.

[34] Otieno PO, Wambiya EOA, Mohamed SM, Mutua MK, Kibe PM, Mwangi B (2020). Access to primary healthcare services and associated factors in urban slums in Nairobi-Kenya. BMC Public Health.

[35] Eskandari M, Abbaszadeh A, Borhani F (2013). Barriers of referral system to health care provision in rural societies in Iran. J Caring Sci.

[36] Naidu A (2009). Factors affecting patient satisfaction and healthcare quality. Int J Health Care Qual Assurance..

[37] Griffiths KM, Crisp DA, Barney L, Reid R (2011). Seeking help for depression from family and friends: a qualitative analysis of perceived advantages and disadvantages. BMC Psychiatry.

[38] Lindsey MA, Joe S, Nebbitt V (2010). Family matters: The role of mental health stigma and social support on depressive symptoms and subsequent help seeking among African American boys. J Black Psychol.

